# Caraway Nanoemulsion Gel: A Potential Antibacterial Treatment against *Escherichia coli* and *Staphylococcus aureus*

**DOI:** 10.3390/gels9030193

**Published:** 2023-03-03

**Authors:** Mohammed H. Alqarni, Ahmed I. Foudah, Alhussain H. Aodah, Faisal K. Alkholifi, Mohammad Ayman Salkini, Aftab Alam

**Affiliations:** 1 Department of Pharmacognosy, College of Pharmacy, Prince Sattam Bin Abdulaziz University, Al Kharj 11942, Saudi Arabia; 2 Department of Pharmaceutics, College of Pharmacy, Prince Sattam Bin Abdulaziz University, Al kharj 11942, Saudi Arabia; 3 Department of Pharmacology & Toxicology, College of Pharmacy, Prince Sattam Bin Abdulaziz University, Al Kharj 11942, Saudi Arabia

**Keywords:** caraway essential oil, nanoemulsion gel, antibacterial, *Escherichia coli*, *Staphylococcus aureus*

## Abstract

Novel antibiotics are needed due to the rise of antibiotic-resistant pathogens. Traditional antibiotics are ineffective due to antibiotic-resistant microorganisms, and finding alternative therapies is expensive. Hence, plant-derived caraway (Carum carvi) essential oils and antibacterial compounds have been selected as alternatives. In this, caraway essential oil as an antibacterial treatment was investigated using a nanoemulsion gel. Using the emulsification technique, a nanoemulsion gel was developed and characterized in terms of particle size, polydispersity index, pH, and viscosity. The results showed that the nanoemulsion had a mean particle size of 137 nm and an encapsulation efficiency of 92%. Afterward, the nanoemulsion gel was incorporated into the carbopol gel and was found to be transparent and uniform. The gel had in vitro cell viability and antibacterial activity against *Escherichia coli* (*E. coli*) and *Staphylococcus aureus* (*S. aureus*). The gel safely delivered a transdermal drug with a cell survival rate of over 90%. With a minimal inhibitor concentration (MIC) of 0.78 mg/mL and 0.78 mg/mL, respectively, the gel demonstrated substantial inhibition for *E. coli* and *S. aureus*. Lastly, the study demonstrated that caraway essential oil nanoemulsion gels can be efficient in treating *E. coli* and *S. aureus*, laying the groundwork for the use of caraway essential oil as an alternative to synthetic antibiotics in the treatment of bacterial infections.

## 1. Introduction

For more than a century, antimicrobial therapy has been an essential part of modern medicine. Nevertheless, the development of antibiotic-resistant pathogens has made this treatment method increasingly difficult [1]. Antimicrobial abuse and misuse, inappropriate dose regimes, and the absence of novel antimicrobial medications all have contributed to the issue [2]. This problem is not new, however, its prevalence is growing as the usage of antimicrobial treatments rises. Yet, as antimicrobial drug usage rises, so does the possibility of resistance development [3]. There is little clarity about where antimicrobial therapy is headed though there are some promising approaches to combating antibiotic resistance. One approach is to develop novel antibiotics that are truly effective against bacteria that have become resistant to existing drugs [4]. Another approach is to find new uses for currently available antibiotics, such as combination treatment, which has been shown to increase antibiotic efficacy [5]. A further approach is to look for antibiotics with a natural origin [6]. This issue must be closely monitored, and effective solutions must be put in place.

Essential oils are concentrated plant extracts with a wide range of chemical constituents recognized to have therapeutic benefits [7,8]. Essential oils have been used medicinally for a very long time, dating back to ancient civilizations such as Egypt, Greece, and Rome. Many diseases, including lung infections, inflammations, gastrointestinal problems, and skin infections, have been treated with essential oils [9]. Essential oils have recently been employed as a natural alternative to synthetic antibacterial agents, especially in the food and cosmetic sectors [10]. The potential of essential oils lies mainly in their antimicrobial properties. It has been shown that essential oils with antimicrobial properties are an effective antimicrobial strategy that prevents the growth of bacteria, viruses, and fungi. The antimicrobial properties of essential oils have been studied extensively. It has been shown that caraway essential oils are effective against many types of bacteria.

Caraway oil is a natural essential oil, extracted from the seeds of the caraway plant (*Carum carvi* L.) and has been traditionally used in culinary and medicinal applications [11]. The main phytoconstituents present in caraway oil include carvone, limonene, and anethole, which are responsible for its characteristic aroma and medicinal properties [12]. It has been traditionally used in various medicinal and culinary applications [13]. Caraway oil has been reported for various pharmacological activities for different disease conditions like spasmetic, carminant, and bacterial effects [14,15]. Caraway essential oil is efficient against a broad spectrum of microorganisms, according to research. *S. aureus*, *E. coli*, and *Salmonella typhimurium* are these microorganisms [16]. Despite its medicinal potential, caraway oil is challenging to employ in formulation development.

One of the major limitations of caraway oil is its strong, pungent aroma. This characteristic can make it difficult to incorporate the oil into formulations without overwhelming other ingredients or altering the desired flavor profile [17]. In addition to these limits, the essential oil is exceedingly perishable and may quickly deteriorate if exposed to light, heat, or air [18]. Caraway oil is a liquid with a strong, pungent aroma, which is hydrophobic in nature. Due to its hydrophobic components, caraway oil is very easily soluble in water and other polar solvents [19]. The low solubility of caraway oil poses a significant challenge for formulation development, particularly in water-based products. The oil droplets tend to form aggregates and remain suspended in the water phase, resulting in poor stability and homogeneity of the final product. This can make it difficult to store and transport the oil, and can further limit its application in formulation development. To maximize caraway oil’s medicinal advantages, further study is required to overcome these restrictions.

Essential oils have poor water solubility and stability, limiting their usage in formulations. Emulsifiers and surfactants may increase caraway oil solubility and stability in water-based products [20]. Emulsifiers, surfactants, and microencapsulations may increase caraway oil solubility and stability in water-based products [21,22]. Research is required to successfully incorporate caraway essential oil into water-based products while preserving its flavor and fragrance. Nanoemulsions with oil droplets in water may solve this issue. These 20–200 nanometer droplets are more stable and distributed than traditional emulsions [23,24]. Nanoemulsion technology makes essential oils stable and water soluble and overcomes these issues [25,26,27]. Nanoemulsions are emulsified droplets of one liquid (caraway essential oil) in another liquid (water). Nanoemulsions with nanometer-sized droplets increase the essential oil’s stability, bioavailability, and permeability. A surfactant or emulsifying agent helps caraway essential oil form an emulsion.

Based on this hypothesis, we developed nanoemulsions of caraway essential oils. Here, with Tween 80 acting as an emulsifying agent, the polyglycerol myristate and polyglycerol monolaurate provide emulsion stability that can help to stabilize the emulsion and prevent the oil and water from separating. After preparation and characterization, the nanoemulsion was incorporated into carbopol gels to enhance the delivery and effectiveness of the essential oils. Carbopol gels are commonly used as topical delivery systems due to of their high viscosity, transparency, and ability to hydrate the skin. By incorporating the nanoemulsion of essential oils into carbopol gels, it can enhance the skin penetration and retention of the essential oils, resulting in better therapeutic effects. This study would also explain how these surfactants generate nanoemulsions and the benefits of employing them in essential oil nanoemulsion technology. It would potentially generate better nanoemulsion products.

## 2. Results and Discussion

### 2.1. Droplet Size, PDI, and Zeta Potential

**Figure 1:**
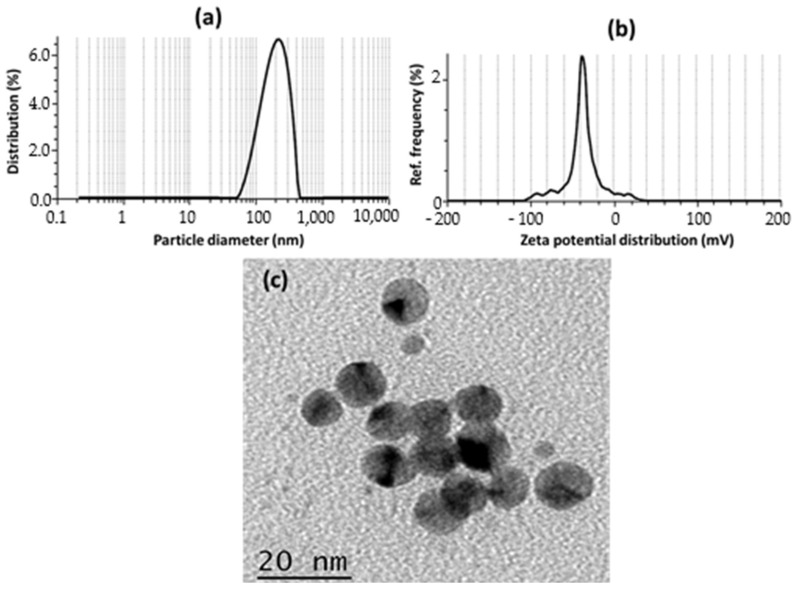
Evaluation of caraway essential oil’s nanoemulsion (**a**) Particle size; (**b**) Zeta potential; (**c**) morphololy analysis of caraway nanoemulsion by TEM.

The results of the study on the droplet size, PDI, and zeta potential of nanoemulsion of essential oils were analyzed and discussed. Nanoemulsion particle size affects its physical, chemical, and application properties. The average nanoemulsion particle size was 100–150 nm, which is standard. The polydispersity index (PDI) was 0.1, suggesting a narrow size distribution and particle uniformity. As illustrated in Figure 1a, a nanoemulsion of essential oils with a particle size of 137 nm and a PDI of 0.1 has small, uniform droplets. A particle size of 137 nm falls within the range of typical nanoemulsions, and the low PDI value of 0.1 indicates that the droplets are well dispersed and have a narrow size distribution. The nanoemulsion’s small droplet size and narrow size distribution may enhance its surface area and stability. Small droplets also improve essential oil bioavailability and effectiveness. Low-PDI nanoemulsions are more stable since the droplets are less prone to aggregate and form bigger particles.

The zeta potential, also known as the electrokinetic potential, of a nanoemulsion of essential oils was measured to be −23 mV, as shown in Figure 1b. This value is indicative of the overall charge of the emulsion droplets and can provide insight into the stability of the system. A negative zeta potential indicates that the droplets are primarily negatively charged, which can lead to repulsion between the droplets and the stability of the emulsion. In contrast, a positive zeta potential would indicate that the droplets are primarily positively charged, which can lead to attraction between the droplets and the instability of the emulsion [28]. Zeta potential is affected by emulsion composition, pH, and stabilizing agents [29]. The negative charge of the droplets in this instance may be attributed to the essential oils in the nanoemulsion, and it is probable that the pH of the system was altered to maintain this charge [30]. In order to keep the emulsion stable, it is also possible that some kind of stabilizing agent was applied. Overall, the zeta potential of −23 mV indicates that this essential oil nanoemulsion is quite stable and will likely continue to be homogeneous over time.

### 2.2. Particle Shape Analysis of Caraway Nanoemulsion by TEM

The morphology of the nanoemulsion of essential oils was thoroughly investigated using TEM. The TEM images revealed that the nanoemulsion droplets had a spherical shape and a mean diameter of 20 nm, as shown in Figure 1c. The photos indicated that the droplets were highly monodispersed, indicating that the emulsification process produced homogeneous droplets efficiently. Furthermore, the TEM images revealed that the droplets had a thin and uniform surfactant film surrounding the droplets, indicating that the droplets were well stabilized. These results were in line with earlier research that demonstrated the tiny size, high monodispersity, and thin surfactant film of nanoemulsion droplets [31]. The stability, bioavailability, and effectiveness of the essential oils in nanoemulsion form depend on these properties. Overall, the TEM examination gave excellent insight into the morphology of the essential oil nanoemulsion and will be helpful in future research on the development and enhancement of nanoemulsion formulations.

### 2.3. Encapsulation Efficiency

This research examined essential oil encapsulation efficiency in nanoemulsion formulations. The percentage of caraway oil encapsulation efficiency in a nanoemulsion was determined to be extremely high at 92% by using the UV method. It was a very stable and uniform combination of water and oil. It has been observed that encapsulating essential oils in a nanoemulsion increases their stability, bioavailability, and therapeutic effectiveness. A nanoemulsion’s small droplets enhance the oil’s surface area, allowing them to enter the skin and interact with the target cells. Encapsulating essential oils in a nanoemulsion has shown promise for formulating natural products with better effectiveness and bioavailability. Research have revealed that surfactant type, essential oil content, and processing conditions may impact encapsulation efficiency [32]. Surfactants and processing conditions may improve encapsulation efficiency and product efficacy. In order to ensure the efficacy and stability of essential oil formulations based on nanoemulsions, it is crucial to take into account the encapsulation efficiency. Encapsulating essential oils in a nanoemulsion for application in food, pharmaceuticals, and cosmetics offers great potential [33]. When assessing the potential of a nanoemulsion for a particular application, the effectiveness of encapsulation is a crucial factor to take into account.

### 2.4. Evaluation of Gels

The evaluation of essential oil nanogels of carbapol 940 for pH was conducted in order to assess the pH-dependent properties of these gels. Carbapol 940 is a commonly used polymeric material in the formulation of nanogels, and its pH-dependent properties have been well established in the literature [34]. However, the addition of essential oils to the carbapol 940 nanogels may affect the pH-dependent properties of the gels [35]. The optimal pH for carbopol gel for transdermal drug delivery is typically around 6.5. This pH range enables the gel to keep its viscosity and stability while also enabling the best possible drug penetration into the skin. The appropriate pH depends on the drug and skin region. The gel may also be made with a skin-friendly pH range of 6–7.

The viscosity of 0.5% carbopol 940 gel in aqueous media is approximately 10,000 to 12,000 centipoise (cP) at room temperature. This viscosity is considered to be a suitable range for transdermal drug delivery as it allows for easy spreading and application on the skin without being too thick or sticky. Overall, the viscosity of 0.5% carbopol 940 gel in an aqueous media is suitable for transdermal drug delivery [36].

The spreadability of a carbopol 940 gel is an important factor to consider when using it as a base for transdermal drug delivery for bacterial treatment. A high spreadability indicates that the gel will easily spread over the skin and allow for more consistent delivery of the drug. In this case, the spreadability of 12.65 ± 0.32 cm at a 0.5% concentration is considered to be good, which may enhance transdermal drug delivery of antibacterial agents.

### 2.5. In Vitro Drug Release Study

**Figure 2:**
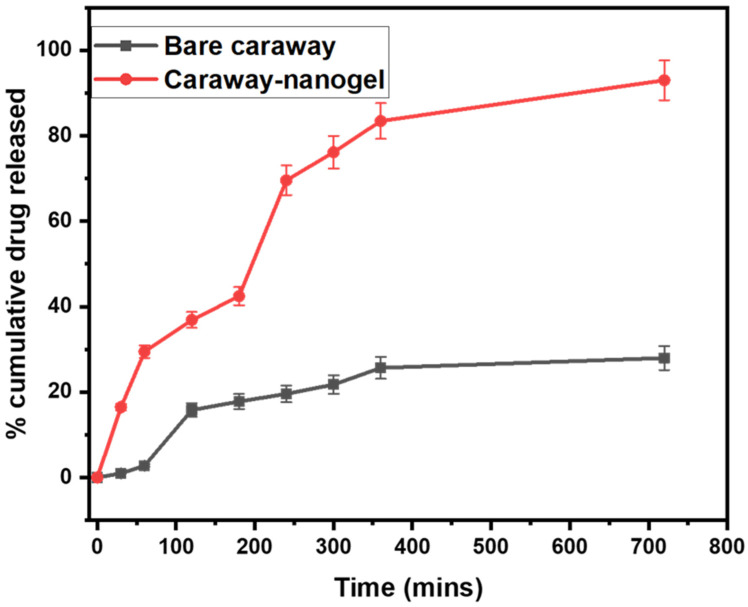
% Cumulative in vitro drug released study of bare caraway essential oil and caraway nanogel in pH 6.8 PBS.

In vitro drug release studies of essential oil nanogels at various timepoints in pH 6.8 can provide valuable information on the potential of these nanogels as transdermal drug delivery systems, as shown in Figure 2. The pH 6.8 condition simulates the pH of the skin, and the release rate of the drugs at different time points can give an indication of the rate at which the drugs are able to permeate through the skin. The release rate was high at early timepoints (30 and 60 min) and this suggests that the nanogels are able to rapidly release the drugs, which may be beneficial for the transdermal delivery of drugs that need to be rapidly absorbed. The drug released was observed to be 16.25 ± 0.8225 (30 min); 36.87 ± 1.84 (120 min), and 69.56 ± 3.468 (300 min) in the case of a caraway nanogel. Whereas in the case of bare caraway essential oil, the drug released was observed to be 0.956 ± 0.44 (30 min); 15.78 ± 1.57 (120 min), and 21.76 ± 3.468 (300 min) as shown in Figure 2. Since nanogel particles are smaller than essential oil particles, essential oil nanogels release more medication in vitro. This increases the surface area and medication release by interacting with the environment.

Further, we carried out the kinetics study as data given in Table 1. The zero-order kinetics observed in the in vitro drug release of essential oil nanogel can be attributed to the fact that the release of the drug is controlled by the diffusion of the active ingredient through the gel matrix, rather than by the degradation or erosion of the gel. The high correlation coefficient (r^2^ = 0.9523) suggests that the release rate is consistent and follows a predictable pattern, which is characteristic of zero-order kinetics. Additionally, the essential oil nanogel may have a high drug loading capacity, which can also lead to a zero-order release pattern. Whereas the in vitro drug release of pure essential oils typically follows first order kinetics with a high r^2^. First order kinetics describes the rate at which a drug is released from a dosage form over time, with the rate being proportional to the concentration of the drug remaining in the dosage form. A high r^2^ of 0.9032 indicates that the data is a very good fit to the first order kinetics model, meaning that the release of the active compounds from the essential oil is well described by this model. Overall, the results of in vitro drug release studies can provide useful information on the potential of essential oil nanogels as transdermal drug delivery systems and can guide the development of these systems for specific therapeutic applications.

### 2.6. Cytotoxicity Study

**Figure 3:**
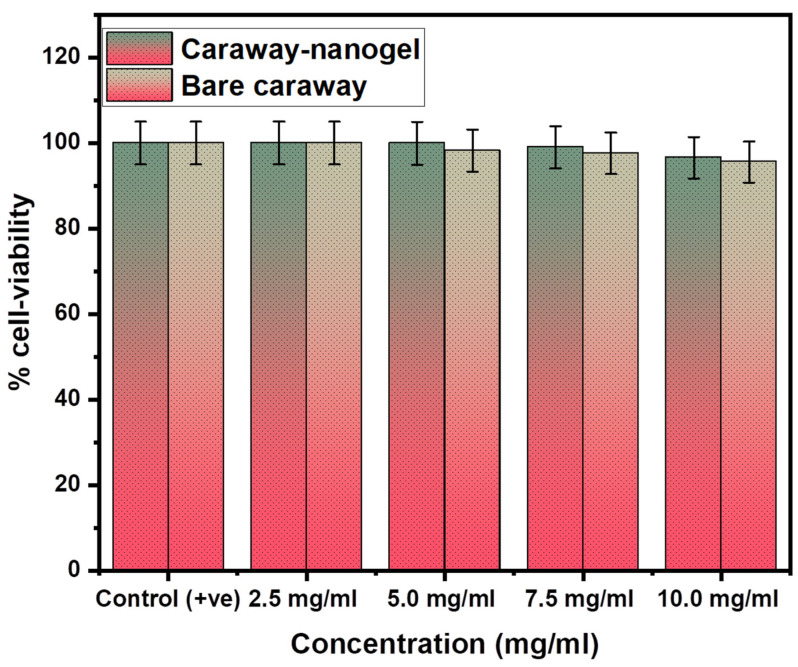
Percent cell-viability of bare caraway essential oil and caraway nanogel in HaCaT cells.

MTT tests evaluated the HaCaT cell safety of essential oils and nanogels. MTT tests cell viability and cytotoxicity. Figure 3 reveals that essential oil and nanogel did not harm HaCaT cells. The study’s essential oil maintained a 90% HaCaT cell viability at all dosages. The essential oil nanogel showed no toxicity on HaCaT cells and had cell survival of over 90% at all dosages. This study found that HaCaT cells were unaffected by essential oils or essential oil nanogels up to 10.0 mg/mL. HaCaT cells use essential oils and nanogels. Essential oils and nanogels seem harmless to human skin cells. Nevertheless, these results only relate to the essential oil and nanogel used in this study. Further research is needed to determine the toxicity of other essential oils and nanogels on human skin cells. MTT cytotoxicity in HaCaT cells suggests that essential oils and essential oil nanogels may be safe for human skin cells, however, further research is required.

### 2.7. Antibacterial Effects

**Figure 4:**
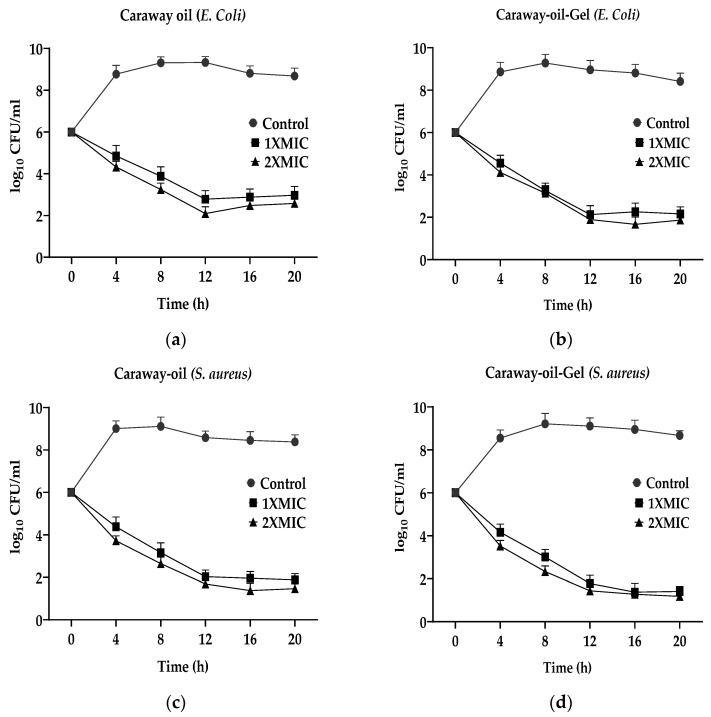
Time-kill assays of *E. coli* and *S. aureus* against caraway oil and caraway oil gel, where (**a**,**b**) are showing the effects of caraway oil and caraway oil-gel against *E-coli* respectively while, (**c**,**d**) are showing the assays of caraway oil and caraway oil-gel against *S. aureus* respectively.

The antibacterial assay of nanogel and essential oil in *Escherichia coli* and *Staphylococcus aureus* was conducted using the well plate method. The results showed that both the nanogel and essential oil were effective in inhibiting the growth of both bacterial strains. In the case of *E. coli*, the nanogel and the essential oil both showed significant inhibition of bacterial growth. Caraway nanogel has a 0.78 mg/mL MIC against *E. coli* and *S. aureus*. Caraway nanogel had 3.125 mg/mL MBC against *E. coli* and *S. aureus*. This demonstrates that the nanogel, at this concentration, is capable of efficiently preventing the development of the bacteria. Caraway nanogel has been shown to be effective against *E. coli* and *S. aureus*. The antibacterial test demonstrated that nanogel and essential oil inhibited *E. coli* and *S. aureus* growth. In both instances, it was determined that the nanogel was more effective than the essential oil. Our data imply the nanogel may be antibacterial against certain bacterial strains. Carvone is an antibacterial chemical that can be found in caraway seeds and is used to make nanogel. It has been shown that carvone has antimicrobial properties [37]. Carvone’s nanogel form is more effective since its smaller particles can penetrate bacterial cell membranes and disrupt cell activity [38]. The literature does not specify the particular mechanism by which the nanogel exerts its antibacterial activity. Several researchers show that plant-based nanoparticles such as caraway nanogel may disrupt bacteria cell membranes, destroying bacteria. Caraway nanogel could treat E. coli and S. aureus infections as an alternative to antibiotics since it inhibits these bacteria at such a low concentration. The nanogel’s mode of action and therapeutic potential require more study. It is crucial to remember that the caraway nanogel MIC value was established under lab conditions, and further research is required to assess its effectiveness in vivo in our next study. Additionally, the antibacterial effect of caraway nanogel against other bacterial strains was evaluated. Comparative data of MIC is given in Table 2. The results of the essential oil and its nanogel time-kill tests against *E. coli* and *S. aureus* are given in Figure 4a–d. The untreated *E. coli* and *S. aureus* data showed growth of more than six and as high as nine (log10 CFU/mL). In comparison, the test group (essential oil and its gel) exhibited a significant drop in the first 8 h. Up until 16 h, everything stayed the same. According to the findings, caraway oil nanogel displays much more than crude essential oil at the 1XMIC and 2XMIC concentrations.

### 2.8. Stability Study

The stability investigation of the nanogel containing essential oils revealed no significant color changes at 1, 7, 14, 21, and 28 days. The nanogel remains stable over time. The nanogel encapsulated essential oils with 90% efficiency. This shows that the nanogel effectively carries and protects essential oils. The nanogel particle size remained consistent at 130–139 nm throughout the investigation. The nanogel is well formed and does not aggregate or disintegrate over time. The stability investigation showed that the essential oil nanogel is a stable and efficient carrier. The nanogel particles stay stable and encase essential oils, protecting them from deterioration. These findings indicate that essential oil nanogels might be used in cosmetics, food, and medicine.

## 3. Conclusions

Antimicrobials cure numerous infectious disorders, revolutionizing medicine. Antibiotic-resistant microorganisms harm antimicrobials. Overuse, misuse, and a shortage of innovative antimicrobials have caused resistance and public health issues. To solve this, researchers are studying new, repurposed, and plant-based antibiotics. Caraway oil has antibacterial potential. Essential oils may prevent antibiotic resistance by preventing bacteria from developing resistance to their bioactive components. This research investigates caraway essential oil’s antibacterial properties using a nanoemulsion gel. We found that using the aforesaid combination instead of synthetic medicines may reduce bacterial infections. The nanoemulsion gel’s efficacy against *Escherichia coli* and *Staphylococcus aureus* shows natural products’ medicinal potential. Herbal treatments may cure infections, however, further research is needed.

This research found caraway oil nanoemulsion gel to be a safe and efficient antibacterial treatment. This is promising for antibacterial treatment and herbal medicine research. This study lays the framework for alternative medications that might treat bacterial illnesses effectively and sustainably. Caraway and other essential oils may replace antibiotics.

Essential oils require additional study. This research has significant limitations. This study’s in vitro trials may not represent in vivo settings, thus further research is required to determine the caraway essential oil nanoemulsion gel’s efficiency. Second, the research only examined the gel’s antibacterial efficacy against two bacterial strains, which is insufficient. Before using the nanoemulsion gel clinically, its long-term safety and stability should be assessed.

## 4. Materials and Methods

Caraway essential oil (≥80%), Tween 80, polyglycerol myristate, and polyglycerol monolaurate was procured from Sigma–Aldrich Chemicals, India. *Escherichia coli* (ATCC-11229) and *Staphylococcus aureus* (ATCC-26923) were collected from the Department of Pharmaceutics, College of Pharmacy, Prince Sattam bin Abdulaziz University, Al-Kharj, Saudi Arabia. All solutions were made with deionized (DI) water. All other chemicals used in this study are of analytical grade and did not require additional purification before use.

### 4.1. Preparation of Nanoemulsion of Caraway

The method for preparing a nanoemulsion of caraway oils involved the use of high pressure homogenization (HPH) as a means of obtaining a more consistent and stable final product. At the beginning of the process, a mixture of deionized water and sorbitol was carefully combined to form the aqueous phase. For the oil phase, Tween 80, polyglycerol myristate, polyglycerol monolaurate, caraway essential oil (100 mg), and medium chain triglycerides were carefully mixed. The two phases were then carefully combined and subjected to intense shearing in a blender for 10 min at an incredibly high speed of 20,000 rpm. This initial mixture was then further processed using a high pressure homogenizer (IKA^®^ WERKE GmBH & Co. KG, Staufen, Germany) and a two stage homogenization valve. Different variations of homogenization pressure (60–110 MPa) and cycle number (5–20) were carefully used to analyze the effects on the properties of the final nanoemulsion and ensure that the final product was of the highest quality [39].

### 4.2. Characterization of Nanoemulsion

#### Droplet Size, Polydispersity Index (PDI), and Zeta Potential (ZP)

Droplet size, PDI, and zeta potential (Anton Paar, Ashland, VA, USA) are important parameters to consider when evaluating the quality of essential oil nanoemulsions in research. Droplet size can be determined using a variety of techniques such as dynamic light scattering (DLS). For DLS, the nanoemulsion was placed in a cuvette and passed through a laser beam, which was then scattered by the droplets. The scattered light was collected by a detector and analyzed using a DLS instrument to determine the average droplet size and size distribution. This method provided information on the average droplet size and size distribution of the nanoemulsion [40]. Next, the polydispersity index (PDI) was calculated using the data obtained from the DLS analysis. The PDI is a measure of the width of the droplet size distribution and can be calculated using the ratio of the standard deviation to the mean droplet size. ZP is a measure of the electrical charge on the surface of the droplets and can be determined using electrophoretic light scattering (ELS) or laser Doppler electrophoresis (LDE) [41]. For this, we prepared a 1% (*w*/*v*) aqueous solution of the nanoemulsion by diluting a small aliquot with distilled water. Measurement of the zeta potential of the nanoemulsion was conducted using a Zetasizer instrument (Anton Paar, Ashland, VA, USA). Based on the results obtained, a conclusion can be drawn about the effect of electrolytes on the zeta potential and stability of the essential oil nanoemulsion.

### 4.3. Particle Shape Analysis of a Caraway Nanoemulsion by TEM

The transmission electron microscopy (TEM) of a liquid sample is a powerful tool for investigating the microstructure and properties of a wide range of materials [42]. The nanoemulsion of caraway essential oils was obtained and placed in a clean container. First, the mixture was then pipetted onto a carbon-coated grid and allowed to sit for a few minutes to allow the sample to adsorb onto the grid. The excess liquid was then carefully removed from the grid using filter paper or a pipette. The grid was then placed in a TEM holder and inserted into the TEM for imaging. The sample was then viewed and analyzed at a high resolution, allowing for the characterization of the particles in the liquid sample. The samples were imaged at different magnifications to study their morphology and size distribution. The entire process should be carried out under a controlled environment to ensure that the sample is not contaminated or damaged during the imaging process.

### 4.4. Percent Encapsulation Efficiency (%EE)

It is typically measured by determining the amount of essential oil present in the nanoemulsion droplets and comparing it to the total amount of essential oil added to the system. The %EE is defined as the percentage of the essential oil that is encapsulated within the nanoemulsion droplets. The percent encapsulation efficiency of the nanoemulsion of essential oils can be calculated using the centrifugation method. In this, the sample was centrifuged at 4000 rpm for 20 min. After centrifugation, the nanoemulsion is separated into two layers; the top layer represents the oil phase, and the bottom layer represents the water phase. The percent encapsulation efficiency can then be calculated using the following equation:

(1) $$\%\;\mathrm{EE}=\frac{\left(Total\;amount\;of\;\mathrm{EOs}\;added-Amount\;of\;free\;\mathrm{EOs}\;in\;supernatent\right)}{Total\;amount\;of\;\mathrm{EOs}\;added}\times 100\ldots \ldots$$

This method allows for the determination of the effectiveness of the encapsulation process and can aid in optimizing the preparation of the nanoemulsion of essential oils for future research.

### 4.5. Incorporation of Nanoemulsion into Carbopol Gels

The incorporation of nanoemulsion into 0.5% carbopol gels was carried out by first preparing the carbopol gel base. To do this, 0.5% carbopol 940 powder was added to distilled water while stirring at a low speed. The mixture was then allowed to hydrate for 10 min before increasing the stirring speed to high for an additional 30 min to ensure proper gel formation. The prepared nanoemulsion was then added to the carbopol gel at a concentration of 2% (*w*/*w*) and mixed until a homogenous gel was formed. We then allowed the mixture to sit for 24 h to allow for proper hydration and formation of the gel. Finally, we checked the consistency and pH of the final product to ensure it met the desired specifications.

### 4.6. Evaluation of Gels

Evaluation of Carbopol gels was performed using several methods to assess the physical and rheological properties of the gels. The texture analysis of a nanogel formulation involves the examination of various physical properties such as rheology, viscosity, pH, and spreadability. These properties are important for understanding the behavior and performance of the nanogel in various applications. This information is crucial for optimizing the nanogel formulation for specific applications and for understanding the underlying mechanisms of its behavior.

The pH: A small amount of the carbopol gel sample was placed in a clean, dry beaker. Deionized water was added to the beaker to create a dilution of the gel. A pH electrode was carefully inserted into the diluted gel solution and allowed to equilibrate for at least 5 min. The pH value was then read directly from the pH meter and recorded [38].

Viscosity measurement: A rheometer was used to measure the viscosity of the carbopol gel. The measurement was repeated at different speeds to obtain a viscosity profile of the sample. The results were then recorded and analyzed to determine the overall viscosity of the carbopol gel [43].

Spreadability: The horizontal plate method was used to check the spreadability of the gel. The ability of the caraway–carbopol nanogel to spread was determined by testing 0.5 g of the gel on a 2 cm diameter circle that was premarked on a glass plate. A second glass plate was used to apply pressure on the gel for 5 min by placing a half-kilogram weight on top. The final size of the circle, after spreading the gel, was then measured to determine the spreadability of the product [44].

### 4.7. In Vitro Drug Release and Kinetics Study

In vitro drug release studies are an important tool for understanding the efficacy and safety of essential oil gels. These studies involve using Franz diffusion equipment to simulate the conditions of the human body, such as pH levels and temperature, to measure the amount of active ingredients released over a certain period of time. The pharmaceutical properties of the gel formulations were evaluated through diffusion cell-based drug release studies. Specifically, the caraway–carbopol nanogel sample (0.5 g) was placed on the membrane and the release study was evaluated under controlled conditions (37 ± 0.5 °C) using a phosphate buffer (pH 6.0) as the receptor medium. A sampling of the receptor medium was conducted at predetermined intervals (15, 30, 60, 120, and 240 min) and aliquots of 2 mL were withdrawn, and subsequently replaced with an equivalent volume of fresh receptor medium. Subsequent analysis of the samples was performed using UV spectrophotometry at a wavelength of 262 nm, with a phosphate buffer serving as the blank control.

The investigation of the temporal dynamics of medication liberation, commonly referred to as drug release kinetics study, aims to determine the rate and mechanism of drug dissolution and/or diffusion from a pharmaceutical formulation. This type of study is crucial in understanding the pharmacokinetics and pharmacodynamics of a drug and can assist in the optimization of dosage regimens and the development of new drug delivery systems. The study typically involves the use of mathematical models, such as zero-order, first-order, and Higuchi equations, to analyze the release data obtained from in vitro or in vivo experiments. The results of the drug release kinetics study can provide valuable information for the design and optimization of pharmaceutical formulations, ultimately improving the therapeutic efficacy and safety of drugs.

### 4.8. Cytotoxicity Study

The procedure for the cell viability assay by the MTT method in HaCaT cells was as follows. The HaCaT cells were seeded into a 96-well plate at a density of 5 × 10^4^ cells per well and incubated overnight at 37 °C in a humidified atmosphere containing 5% CO_2_. Following incubation, the cells were treated with various concentrations of a test compound for 24 h. After treatment, the medium was removed and replaced with 200 µL of fresh medium containing MTT (3-(4,5-dimethylthiazol-2-yl)-2,5-diphenyltetrazolium bromide) at a final concentration of 0.5 mg/mL. The cells were then incubated for 4 h at 37 °C in a humidified atmosphere containing 5% CO_2_. Following incubation, the medium containing MTT was removed and replaced with 200 µL of DMSO (dimethyl sulfoxide) to dissolve the formazan crystals formed by the viable cells. The absorbance of the formazan product was measured at 570 nm using a microplate reader. The cell viability was calculated as a percentage of the absorbance values obtained from the treated cells compared to the untreated control cells.

### 4.9. Antibacterial Activity

The antibacterial assay of nanogel in *E.coli* and *S. aureus* was performed using the 96-well plate method. First, a series of concentrations (0.19503 to 25 mg/mL) of the essential oil and its nanogel were prepared in 2% dimethyl sulfoxide (DMSO). Then, overnight cultures of both *E. coli* and *S. aureus* were grown in a nutrient broth at 37 °C and diluted to a final concentration of 1 × 10^6^ CFU/mL. Then, 100 μL of the bacterial suspension were added to each well of a 96-well plate, and 100 μL of the different nanogel concentrations were added to the corresponding wells. The plate was then incubated at 37 °C for 24 h. After incubation, the absorbance of the bacterial suspensions was measured at 600 nm using a microplate reader. The minimum inhibitory concentration (MIC) was determined as the lowest concentration of the nanogel and the minimum bactericidal concentration (MBC) was determined to be the lowest concentration of nanogel required to kill a particular bacterium that resulted in a significant reduction in bacterial growth as compared to the control wells without the nanogel. The results of the assay demonstrated that the nanogel effectively inhibited the growth of both *E. coli* and *S. aureus* at concentrations lower than the MIC. The cell viability (time-kill) assays were performed following the previous method [45].

### 4.10. Stability Study

A stability study of nanogel was conducted for one month in order to evaluate the physical and chemical properties of the gel over time. The study was performed in a controlled environment at room temperature (25 °C) and relative humidity (50%) to minimize any external factors that may affect the stability of the gel. At the beginning of the study, the nanogel samples were prepared and characterized for their color change, encapsulation efficiency, and particle size. These initial measurements were used as a reference for the subsequent evaluations. The samples were then stored in sealed containers and checked for any changes in their physical properties, such as size, shape, and zeta potential, at 1, 7, 14, 21, and 28 days. Additionally, the chemical properties of the nanogel were evaluated by measuring the pH, viscosity, and spreadability.

## Figures and Tables

**Table 1:** Kinetic model for bare caraway essential oil and caraway nanogel after dissolution study.

Formulation	Zero Order	Higuchi	First Order	Kors–Peppas	Hixson–Crowell
Bare caraway essential oil	0.893	0.8832	0.9032	0.6593	0.8991
Caraway nanogel	0.9523	0.9395	0.9401	0.7502	0.9532

**Table 2:** Comparative MIC determination of the different compounds against *E-coli* and *S. aureus*.

Sample	*E-coli* (mg/mL)		*S. aureus* (mg/mL)	
	MIC	MBC	MIC	MBC
Bare caraway essential oil	1.56	3.125	1.56	3.125
Caraway nanogel	0.78	1.56	0.78	1.56
Nanogel	NA		NA	

## Data Availability

All data generated or analyzed during this study are included in this published article.
